# The effect of employee mindfulness in the new media industry on innovative behavior: The chain mediating role of positive emotion and work engagement

**DOI:** 10.3389/fpsyg.2022.976504

**Published:** 2022-11-14

**Authors:** Ting Yue Kuang, Yue Hu, Yan Lu

**Affiliations:** ^1^Faculty of Business, City University of Macau, Macau, Macao SAR, China; ^2^Institute for Research on Portuguese-Speaking Countries (IROPC), City University of Macau, Macau, Macao SAR, China

**Keywords:** employee mindfulness, positive emotion, work engagement, innovative behavior, new media industry

## Abstract

Mindfulness has long been concerned and emphasized by scholars in the field of psychology, but there is still a lack of research on mindfulness in management in China. In this study, a questionnaire survey was conducted among 483 employees in the new media industry in Beijing, Shanghai, Guangzhou, Shenzhen, and Hangzhou, China. After modeling and analysis, it was found that employee mindfulness has a positive influence on innovative behavior. Employee mindfulness and innovative behavior are mediated by positive emotions. Employee mindfulness and innovative behavior are mediated by work engagement. Employee mindfulness and innovative behavior are mediated by a chain of positive emotions and work engagement. Enterprise managers should improve the level of mindfulness of employees in the new media industry through mindfulness training and courses for mindfulness training, create an organizational environment that can arouse positive emotions and improve the positive emotions of employees, pay attention to arousing the enthusiasm of the staff, and promote the innovative behavior of staff while enhancing work engagement.

## Introduction

In the 21st century, global information technology represented by the Internet is changing with each passing day, promoting the rapid development of emerging media, and media communication soon enters the era of new media (Chen and He, 2022). As an important industry for China’s future development, new media industry is constantly integrating into various fields of China’s social economy and people’s livelihood. As of December 2021, the number of Internet users in China reached 1.032 billion, and the Internet penetration rate was as high as 73%, providing basic impetus for the sustainable development of China’s new media industry (Hu et al., 2022).

With the rapid development of China’s new media industry, people’s choice of new media products is showing a diversified trend. In order to meet the needs of the public, new media practitioners not only need to have various professional skills, but also need to have the ability to innovate. Employees in the new media industry inevitably encounter various high demands in their daily work (Dediu et al., 2018), which greatly reduce their innovative behavior and willingness while affecting their mood as well as their work status (Martín-Hernández et al., 2020). The innovative behavior of employees is essential for the sustainability of the organization (De Jong and den Hartog, 2010; Martín-Hernández et al., 2020). How to cultivate, develop, stimulate, and utilize the innovative ability of employees in the new media industry is an issue that managers should pay attention to.

Mindfulness is a relatively new concept in psychological research (Keng et al., 2011), which refers to “awareness and observation of the present moment without responsiveness or judgment” (Glomb et al., 2011). As the study progressed, researchers found that mindfulness also plays a huge role in the field of management (Good et al., 2016), especially in enhancing employees’ innovative behavior. When mindfulness helps employees to slow down and concentrate (Hensley, 2020), it enables people to retain useful and relevant information, which, in turn, increases the ability of individuals to generate new ideas (Montani et al., 2018) and innovative behavior (Khan and Abbas, 2022). In addition, studies have found that positive emotions (Wang Z. et al., 2021) and work engagement (Montani et al., 2020) also play an important role in enhancing employees’ innovative behavior. On the one hand, employees with positive emotions have a wide range of ideas (Isen, 1993) and are not afraid of the risks and challenges brought by innovation (Schwarz and Bohner, 1996), so they are more likely to carry out innovative activities. On the other hand, employees with high work engagement are more focused on their work (Amabile, 1997), and will stimulate their initiative and put more ideas into action (Pieterse et al., 2010), thus stimulating their innovative behavior. Mindfulness as a trait has a significant positive impact on regulating employees’ emotions (McLaughlin et al., 2019) and enhancing employees’ work engagement (Leroy et al., 2013). According to resource conservation theory, mindfulness is found to be beneficial in helping employees store, perceive, and utilize individual resources, and when employees’ own resources are increased, it stimulates and enhances positive emotions (Du et al., 2019; Alhawatmeh et al., 2022) as well as work engagement (Brown and Ryan, 2003; Leroy et al., 2013), thus promoting the occurrence of innovative behaviors of employees.

Currently, the literature researching the role between employee mindfulness and innovative behavior is scarce. The existing literature mostly starts the analysis from the aspects of creative process engagement (Khan and Abbas, 2022) and subjective well-being (Liu et al., 2020a,b). Positive emotions and work engagement are important internal factors to stimulate employee innovative behavior (Amabile et al., 2005; Kim and Park, 2017; Jung and Yoon, 2018), but almost no scholars have analyzed the mechanism of the interaction between mindfulness and innovative behavior, and no one has taken employees in the new media industry as the research object. The vigorous development of the new media industry is inseparable from innovation. Therefore, this study explores the relationship among employee mindfulness, positive emotion, work engagement, and innovative behavior through empirical research, which is of great significance to stimulate the innovation ability of employees in the new media industry and the innovation level of enterprises.

## Literature review and hypotheses

### Employee mindfulness and innovative behavior

Mindfulness, originally derived from the meditation training of Buddhism, mainly refers to the intensity of individual attention (Jacobs and Blustein, 2008). Research on mindfulness is generally divided into trait mindfulness and state mindfulness (Randal et al., 2015). State mindfulness can be changed due to internal and external environmental stimuli and lasts for a short time (Liu et al., 2022a), and this state is not fixed (Said and Tanova, 2021). Trait mindfulness is a tendency to remain stable over time, that is, individuals actively and consciously pay attention to the surrounding environment and remember relevant useful information, so that the mind can focus more on the objects related to the goal and be more determined (Wang X. et al., 2021). The improvement of innovative behavior of employees is exactly the stimulation of trait mindfulness. This study mainly analyzes and studies long-term and sustained behavioral effects, and therefore investigates mindfulness as a trait.

Innovation refers to the new integration of the knowledge owned by individuals and the new knowledge acquired, which can enable enterprises to obtain new opportunities more effectively and rationally utilize existing opportunities (Matzler et al., 2013). Innovative behavior often occurs at the individual level (Nonaka and Takeuchi, 1995). When individuals are creative, they can not only complete their work better, but also help enterprises gain competitive advantages in an uncertain environment (Wang et al., 2017). As a trait, employee mindfulness is an important factor affecting their innovative behavior (Mulligan et al., 2021). On the one hand, mindfulness can improve individual alertness and cognitive flexibility (Good et al., 2016; Van gelderen et al., 2019). The flexible cognition generated by mindfulness supports adaptation by generating novel ideas and responses (Good et al., 2016), while alertness helps individuals maintain extensive external attention and capture important information, thereby reducing error rate and enhancing their ability to generate new ideas (Dane and Brummel, 2014). On the other hand, mindfulness reduces automatic responses and enhances attentional breadth (Dane and Rockmann, 2020). When mindfulness helps individuals to focus (Hensley, 2020), it will make them retain useful and relevant information, so as to have a deeper understanding of the problem, and then generate more flexible new ideas (Montani et al., 2018; Khan and Abbas, 2022).

According to social cognitive theory, individual psychological traits can influence individual behavior (Bandura, 1986). Mindfulness is an individual’s internal perception of inner changes or inner emotional changes caused by external stimuli (Brown and Ryan, 2003). Innovation behavior is a phased process, including not only the generation of new ideas, seeking support for individual innovation ideas, but also the implementation of new ideas (Scott and Bruce, 1994). Previous studies have confirmed that mindfulness positively affects innovative behavior (Jobbehdar Nourafkan et al., 2022; Khan and Abbas, 2022). In conclusion, we make the hypotheses:

*H1*: Employee mindfulness has a significant positive impact on innovative behavior.

### Employee mindfulness, positive emotion, and innovative behavior

Emotion is a physiological arousal state and a cognitive state corresponding to this arousal state (Schachter and Singer, 1962). People’s emotions in the face of various things in life reflect people’s evaluation of these things (Diener et al., 1999). Emotions include both positive and negative emotions (Watson and Tellegen, 1985). At present, negative emotions have been studied by many psychologists, and some scholars say that studying positive emotions is also very important (Seligman and Csikszentmihalyi, 2014). Positive emotion is often a transient emotional experience, which is an individual’s response to meaningful events in life (Fredrickson, 2001). Individuals with high levels of positive emotions show more flexible thinking, faster conversion speed, and accept a wider range of behavioral choices (Kahn and Isen, 1993). In essence, negative and positive emotions are incompatible, but experiments have shown that positive emotions can reduce or eliminate reactions caused by negative emotions (Fredrickson, 1998; Fredrickson, 2001). At the same time, people can improve their mental resilience and mental health by cultivating positive emotions at appropriate times, thus promoting their physical health (Fredrickson, 2000). Mindfulness is not only a way of emotion regulation, but also a kind of emotion regulation ability. Previous studies have shown that mindfulness is closely related to emotion regulation (Lutz et al., 2014; McLaughlin et al., 2019; Liu et al., 2022b), in which there is a spiraling process between mindfulness and positive emotions, that is, by improving individual mindfulness, people with positive emotions will generate more positive emotions in the future, and the increase of positive emotions predicts more mindfulness (Du et al., 2019).

Numerous studies have shown that positive emotions positively predict employees’ innovative behavior (Madrid et al., 2014; Zhou et al., 2014; Wang Z. et al., 2021; Caniels et al., 2022; Zhou et al., 2022). The broaden-and-build theory of positive emotions suggests that positive emotions can expand the scope of an individual’s immediate thinking actions by constructing more enduring personal resources such as psychological, social, physical, and intellectual resources to provide more sustainable adaptive benefits for the development provided by the individual (Fredrickson, 2001). On the one hand, individuals under the influence of positive emotions can flexibly adjust their cognitive state and improve the level of thinking activity (Madrid, 2020), thus generating extensive and diverse ideas, which is often the source of individual innovative behaviors (Isen, 1993). On the other hand, positive emotions, as a comfortable state, can make individuals not afraid of risks and challenges in the process of innovation, show amazing expansion of thinking in urgent problems (Zhou et al., 2022), and enhance the exploration of new procedures, so as to carry out innovative activities (Schwarz, 1990; Schwarz and Bohner, 1996). In addition, positive emotions also expand the attention range of individuals (Fredrickson and Branigan, 2005), which enables individuals to guide their behavioral responses more effectively. When individuals are in a state of positive emotion, they are more likely to choose more challenging goals (Cardon et al., 2009) and motivate them to act in an innovative way and make more efforts (Caniels et al., 2022).

According to the broaden-and-build theory of positive emotions (Fredrickson, 2001), mindfulness promotes the generation of positive emotions and further improves the cognitive flexibility and attention of individuals, thus expanding the scope of instant thinking and action of individuals and generating innovative behaviors. Previous studies have confirmed the mediating effect of positive emotions between mindfulness and creativity (Chen et al., 2022). Based on this, we propose the following three hypotheses:

*H2*: Positive emotions play a mediating role in the relationship between employee mindfulness and innovative behavior.

*H2a*: Employee mindfulness positively affects positive emotions.

*H2b*: Positive emotions positively affect innovative behavior.

### Employee mindfulness, work engagement, and innovative behavior

Work engagement is an active state of work, characterized by concentration, energy, and dedication (Schaufeli et al., 2002). As a good working attitude (Rahman and Karim, 2022), work engagement not only affects organizational commitment (Ahmad and Gao, 2018), employee job satisfaction (Van Tuin et al., 2021), and turnover intention (Cao and Chen, 2021), but also has an important influence on organizational citizenship behavior (Ismael et al., 2022) and innovative behavior (Bakker et al., 2014), which is one of the basic factors influencing the results and attitudes of various behaviors in the workplace (Han et al., 2021).

Innovation is a dynamic and complex process, and the stimulation of innovative behavior requires individuals not only to possess certain professional knowledge, ability, and motivation, but also to invest a lot of energy and time in continuous trial and error and improvement (Amabile, 1997). It has been found that the employee’s work engagement can promote innovative behavior (Hakanen et al., 2008; Zhang and Bartol, 2010). On the one hand, employees with high work engagement are more focused on their work, and they will continue to finish their work when faced with difficulties or bottlenecks, such concentration and persistence are the sources of promoting their creativity (Amabile, 1997). On the other hand, high work engagement will reduce employees’ stress (Britt et al., 2001) and promote an increase in positive emotions (Hakanen et al., 2008), which will further stimulate their initiative and put more ideas into action. Thus, more innovative behaviors are generated (Pieterse et al., 2010). Mindfulness can enhance engagement in activities or work by increasing the vividness and clarity of an individual’s experience (Brown and Ryan, 2003), helping employees to “accept” what is known and change their perspective (Shapiro et al., 2006; Carmody et al., 2009), and making employees more focused on their work and maintain interest in it, thus stimulating employees’ innovative behavior (Sonnentag, 2003).

Conservation of Resource Theory holds that individuals are motivated to use and invest resources (Hobfoll, 1989). When initial resources are abundant, individuals will make full use of existing resources and put them into subsequent activities or work (Hobfoll, 2011; Halbesleben et al., 2014). However, when initial resources are relatively scarce, individuals are vulnerable to the risk of resource loss, which leads to more cautious use of resources. Mindfulness is an internal resource possessed by individuals (Grover et al., 2017; Montani et al., 2018; Fisher et al., 2019). It can help individuals store energy and enhance their awareness of other resources so that individuals can perceive more alternative resources (Kroon et al., 2015) and make good use of resources in subsequent activities (Shapiro et al., 2006; Good et al., 2016). Therefore, when employees experience high mindfulness, they will devote more resources to their work, making them more focused, and the resulting high level of work engagement will further promote the occurrence of innovative behavior. Previous studies have confirmed that mindfulness can enhance individual concentration and immersion and has a positive correlation with work engagement (Brown and Ryan, 2003; Leroy et al., 2013). Meanwhile, work engagement has a promoting influence on increasing employee innovative behavior (Rich et al., 2010; Aryee et al., 2012; Kim and Park, 2017; Jung and Yoon, 2018), but fewer studies have used work engagement as a mediator to study employee mindfulness and innovative behavior. Thus, we propose the following three hypotheses:

*H3*: Work engagement plays a mediating role in the relationship between employee mindfulness and innovative behavior.

*H3a*: Employee mindfulness positively affects work engagement.

*H3b*: Work engagement positively affects innovative behavior.

### Positive emotion and work engagement

Work engagement is a pleasant state of mind associated with one’s job (Schaufeli and Bakker, 2004), which essentially contains a series of positive emotions (Ouweneel et al., 2012b). The broaden-and-build theory for positive emotions holds that positive emotions can not only bring instantaneous expansion of individual thinking activities, but also help individuals build long-term and abundant individual resources (Fredrickson, 2001), and the increase in individual resources is usually caused by positive emotional experience. This side effect will increase the motivation, initiative, and enterprising spirit of the individual (Schaufeli et al., 2002). When employees are in a positive emotional state, they will positively evaluate and experience their work and surrounding environment, thus stimulating the behavior of absorbing new information, experience, and exploration, and in this process, expand themselves and become more engaged in their work to achieve their goals (Forgas, 1995). Therefore, individuals with positive emotions are more inclined to increase their personal and job resources and devote them to work (Ouweneel et al., 2012a). Scholars have confirmed that positive emotions have an important positive influence on employees’ work engagement (Leavitt et al., 2019; Wu and Wu, 2019; Ameer and Zubair, 2020; Yuan et al., 2020).

In addition, according to the conservation of resource theory, mindfulness is conducive to the storage, perception, and utilization of individual resources, and when employees’ own resources are increased, it will stimulate and enhance employees’ positive emotions (Du et al., 2019; Alhawatmeh et al., 2022) and work engagement (Brown and Ryan, 2003; Leroy et al., 2013), which will promote the occurrence of employee innovative behavior.

Hence, we propose the following hypothesis:

*H4*: Positive emotions positively affect work engagement.

*H5*: Positive emotion and work engagement play a chain mediating role between employee mindfulness and innovative behavior.

**Figure 1:**
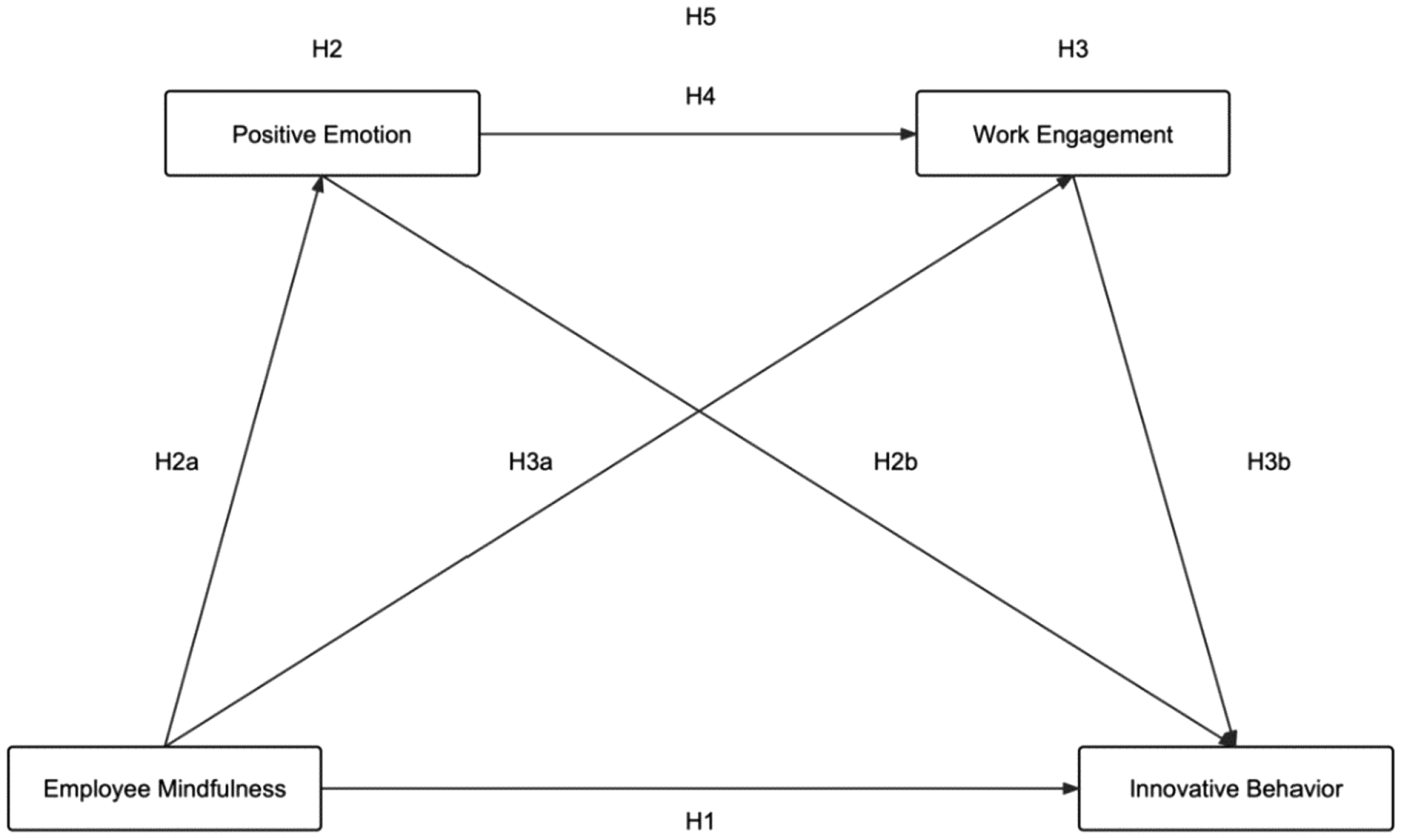
Research model and hypotheses.

To summarize, Figure 1 shows the research model and hypothesis established in this study.

## Methodology

### Participants and collection

New media industry refers to related industries that rely on emerging technologies (digital technology, mobile communication technology, etc.) and take emerging media such as mobile phone, Internet, interactive TV media, and new media such as mobile TV and building TV as carriers (Gong and Weng, 2010). In this study, employees in the new media industry are defined as employees engaged in relevant enterprises (such as Weibo, WeChat, webcast, short video, etc.) that use Internet technology as the media of information dissemination. According to China Statistical Report on Internet Development, Beijing, Shanghai, Guangzhou, Shenzhen, and Hangzhou are the five cities with good economic development and the most listed Internet companies (China Internet Network Information Center, 2021). Research has found that economic development and Internet penetration rate are both important driving forces to promote the development of new media industry (Guo and Fang, 2022). Therefore, Beijing, Shanghai, Guangzhou, Shenzhen, and Hangzhou were selected as sample collection sites in this study.

Questionnaire Star,^1^ one of the largest questionnaire collection platforms in China, is a professional survey distribution platform. In the collection of questionnaires, in order to ensure the authenticity of the questionnaire recovery, the questionnaire must be filled by a real-name user (the questionnaire fillers are informed in advance that it is only for academic research and will not involve any privacy), and each user can only fill in the questionnaire once. The participants of the questionnaire responded more positively to the collection of the online questionnaire (Huang et al., 2016), and the online questionnaire can not only expand the sample size, but also improve the reliability and validity of the sample (Cao et al., 2021). Therefore, this study adopts online questionnaire to conduct convenient sampling of employees in the new media industry in these five cities. Convenient sampling is easy to implement and has the advantage of saving time and cost. Previous studies have also confirmed the effectiveness of convenient sampling based on online questionnaire (Zhu et al., 2022).

The questionnaire consists of two parts. The first part contains four scales of employee mindfulness, positive emotion, work engagement, and innovative behavior, and the second part contains relevant information of the questionnaire fillers. A total of 550 questionnaires were collected in this study, of which 483 were valid, with an effective rate of 87.82%. There were 310 females (64.2%) and 173 males (35.8%). In terms of age, the number of people aged 26–35 was the largest (36.2%). In terms of marital status, 52.8% of employees were married. In terms of education, the largest number of people had a bachelor’s degree (48%). The number of people who had worked for more than 10 years was the highest in terms of working years (34.2%). In terms of jobs, the proportion of ordinary employees (Ordinary employees are employees in non-management positions) was up to 60.7% (see Table 1 for details).

**Table 1:** Demographic characteristics (*N* = 483).

Variables		Frequency	%
Gender	Female	310	64.20%
	Male	173	35.80%
Age	18–25	130	26.90%
	26–35	175	36.20%
	36–45	132	27.30%
	46–55	37	7.70%
	Older than 55	9	1.90%
Marital status	Married	255	52.80%
	Unmarried	222	46.0%
	Others	6	1.20%
Educational background	Graduates of high school or technical secondary school or below	26	5.40%
	Junior college graduates	94	19.50%
	Bachelor	232	48.00%
	Master or above	131	27.10%
Years of working	<1	94	19.50%
	1–3	92	19.00%
	4–5	48	9.90%
	6–10	84	17.40%
	>10	165	34.20%
Job title	Ordinary employee	293	60.70%
	Grass-roots manager	78	16.10%
	Middle manager	79	16.40%
	Senior manager	33	6.80%

### Measures

#### Mindfulness attention awareness scale

In this study, the single-dimension scale of mindful attention awareness developed by Brown and Ryan (2003) was adopted, which has 15 items in total. Through confirmatory factor analysis, 6 items with factor loading coefficients lower than 0.6 were deleted, and there remained 9 items, making the scale more suitable for this study (for example, items include “I find it difficult to stay focused on what’s happening in the present.”). This scale was self-rated by employees, and the Likert 5-point scale was used to score the questionnaire (1 = completely inconsistent, 5 = completely consistent). All the questions are reverse scored. Therefore, conversion was conducted in the empirical analysis of this study.

#### Positive emotion scale

Watson et al. (1988) created an emotion scale that encompasses both positive and negative emotions. In this study, 10 words of positive emotion were selected, including Enthusiastic, Excited, and Determined. A 5-point Likert scale was used to score the questionnaire (1 = almost none, 5 = very much).

#### Work engagement scale

The work engagement scale prepared by Schaufeli et al. (2006) was adopted in this study, which included three dimensions of vitality, dedication, and concentration, with three questions in each dimension and nine items in total. Words such as “I am enthusiastic about my job, I am immersed in my work” were scored using a five-point Likert scale (1 = strongly disagree, 5 = strongly agree).

#### Innovative behavior scale

Scott and Bruce (1994) developed the Innovation Behavior Scale, which has 6 items(for example, “Promotes and Champions ideas to others.”). The questionnaire adopted A 5-point Likert scale scoring (1 = strongly disagree, 5 = strongly agree).

### Data analysis

In this study, SPSS 23.0, PROCESS 3.3, and Amos 23 were used to analyze the data. AMOS was mainly used in model testing, while SPSS was mainly used in descriptive statistical analysis and regression analysis. PROCESS was used to test the mediating effect of the model.

### Control variables

We controlled for gender, age, marital status, educational background, working years, and job title because demographic variables will influence employees’ innovative behavior (Tang et al., 2017).

## Results

### Test of common method bias

To reduce the common method bias, we first introduced the purpose of this study to the questionnaire participants. Secondly, to reduce the concern of the participants, we explained to the questionnaire participants that there is no right or wrong and ensured that all the answers were anonymous. Finally, Harman single-factor test was used to test the collected data to avoid the common method bias. The results show that the eigenvalues of five factors are greater than 1, and the maximum factor variance explanation rate is 39.241% and <40% (Podsakoff et al., 2003), which indicates that there is no serious common method bias in this study.

### Descriptive statistical analysis

In this study, the control variables (gender, age, marital status, educational background, working years, and job title) had no effect on the current research hypothesis. The correlation coefficient, mean value, and standard deviation of each variable are shown in Table 2. The results show that employee mindfulness (EM) is positively correlated with positive emotions (PE; *r* = 0.368, *p* < 0.01), work engagement (WE; *r* = 0.330, *p* < 0.01), and innovative behavior (IB; *r* = 0.369, *p* < 0.01). PE are positively correlated with WE (*r* = 0.651, *p* < 0.01) and IB (*r* = 0.541, *p* < 0.01). There is a positive correlation between WE and IB (*r* = 0.569, *p* < 0.01).

**Table 2:** Correlation coefficient, mean value, and standard deviation among variables.

	M	SD	1	2	3	4
1.Employee mindfulness	3.76	0.80	**0.902**			
2. Positive emotion	3.54	0.68	0.368^**^	**0.934**		
3. Work engagement	3.49	0.75	0.330^**^	0.651^**^	**0.934**	
4. Innovative behavior	3.70	0.65	0.369^**^	0.541^**^	0.569^**^	**0.893**

N = 483; The Cronbach’s α coefficient is in bold.

** *p* < 0.01.

### Model inspection

CFA was carried out using Amos 23. Table 3 shows the results. The model fitting indexes are χ2 = 1497.838, df = 519, χ2/df = 2.886, CFI = 0.914, TLI = 0.907, RMSEA = 0.063. All the indicators are within the acceptable limits, so the four-factor model is the most appropriate (Table 3).

**Table 3:** CFA.

Model	χ2	df	χ2/df	CFI	TLI	RMSEA
Four-factor model (EM, PE, WE, IB)	1497.838	519	2.886	0.914	0.907	0.063
Three-factor model (EM, PE and WE, IB)	2861.117	524	5.460	0.794	0.779	0.096
Two-factor model (EM and PE, WE and IB)	4158.209	526	7.905	0.679	0.658	0.120
Single-factor model (EM and PE and WE and IB)	5229.820	527	9.924	0.585	0.558	0.136

*N* = 483. EM, Employee mindfulness; PE, Positive emotion; WE, Work engagement; IB, Innovative behavior.

### Direct effect test

SPSS 23.0 was used for regression analysis based on good model fitting, and the results are reported in Table 4. EM positively affects IB (*β* = 0.300, *p* < 0.001), H1 is supported; EM positively affects PE (*β* = 0.311, *p* < 0.001), H2a is supported; PE positively affects IB (*β* = 0.521, *p* < 0.001), H2b is supported; EM positively affects WE (*β* = 0.309, *p* < 0.001), H3a is supported. WE positively affects IB (*β* = 0.495, *p* < 0.001), H3b is supported. PE positively affects WE (*β* = 0.721, *p* < 0.001), H4 is supported.

**Table 4:** Studies the direct impact of pathways.

Path	β	SE	*P*-value
H1: EM → IB	0.300	0.035	0.000
H2a: EM → PE	0.311	0.036	0.000
H2b: PE → IB	0.521	0.037	0.000
H3a: EM → WE	0.309	0.040	0.000
H3b: WE → IB	0.495	0.033	0.000
H4: PE → WE	0.721	0.038	0.000

EM, Employee mindfulness; PE, Positive emotion; WE, Work engagement; IB, Innovative behavior.

### Indirect effect test

The indirect effect was tested using the PROCESS 3.3 plug-in (Table 5). The indirect effect of EM on IB through PE is 0.0759, and the confidence interval of 95% is [0.0337, 0.1276] (excluding 0), showing that PE has a significant mediating effect, and H2 is supported. EM has a 0.0299 indirect effect on IB through WE, and the confidence interval of 95% is [0.0068, 0.0601] (excluding 0), showing that WE has a significant mediating effect, and H3 is supported. EM has a 0.0644 indirect influence on IB through PE and WE, and the confidence interval 95% is [0.0373, 0.0965] (excluding 0), showing that PE and WE are significant in the chain mediation of EM and IB, and H5 is supported.

**Table 5:** Indirect effects of research pathways.

	Effect	BootSE	BootLLCI	BootULCI
TOTAL	0.1702	0.0305	0.1138	0.2320
EM → PE → IB	0.0759	0.0240	0.0337	0.1276
EM → WE→ IB	0.0299	0.0137	0.0068	0.0601
EM → PE → WE → IB	0.0644	0.0151	0.0373	0.0965

EM, Employee mindfulness; PE, Positive emotion; WE, Work engagement; IB, Innovative behavior.

## Discussion

### Theoretical significance

First, most previous studies on workplace employees have focused on state mindfulness (e.g., Olafsen, 2017; Said and Tanova, 2021), which focuses on the antecedents of state mindfulness. However, there are few studies on employee trait mindfulness. Trait mindfulness is a personal trait that focuses more on how it affects work outcomes as a predictor (Wang X. et al., 2021). This study confirmed that trait mindfulness is initiated by a top-down mechanism (Wang Z. et al., 2021), in which employees are conducive to the occurrence of innovative behavior. This study has helped spark researchers’ interest in the effects of trait mindfulness on employee behavior and attitudes.

Secondly, previous antecedent studies on employees’ innovative behavior have focused on external factors, such as external environment, leadership influence, leadership style, and the exchange relationship between leaders and subordinates (Dhar, 2016; Leitch and Volery, 2017; Hoang et al., 2022). However, these studies ignore the vulnerability of external factors to other factors (such as the closeness of the relationship between enterprise managers and employees; Kim et al., 2021) and the importance of internal factors. Studies show that positive emotions and work engagement, as positive mental states of individuals, play an important role in improving their innovative behavior. This study helps us to understand the influencing factors and influencing mechanism of employee innovation behavior from the perspective of internal factors of employees, and further enriches the theoretical development of innovative behavior.

Finally, although previous studies have explained the importance of mindfulness in improving innovative behavior among employees (Jobbehdar Nourafkan et al., 2022; Khan and Abbas, 2022), but very little is known about how it works. This study uses a research model to clarify how mindfulness improves the operating mechanism of employees’ innovative behavior, thus helping enterprises gain competitive advantages. This study supports the theory of resource conservation, in which individuals are motivated to use and invest resources. Studies have shown that mindfulness, as an individual trait, can help employees store resources and enhance the perception, discovery, and utilization of other resources (Good et al., 2016), by stimulating employees’ positive emotions, more resources will be put into work, which will greatly enhance employees’ focus and cognitive flexibility, so as to promote the occurrence of innovative behavior. Few scholars have studied the chain mediating effect of positive emotions and work engagement on the relationship between mindfulness and employees’ innovative behavior, let alone employees in the new media industry. This study not only broadens the research scope of employee mindfulness, but also deeply analyzes the mechanism of employee mindfulness in the new media industry on innovative behavior, which has important theoretical contributions.

### Practical significance

The results of this study have important practical significance for improving the innovative behavior of employees in the new media industry and can provide some constructive suggestions for human resource management practices in the new media industry. First, employee mindfulness has a positive effect on improving employees’ innovative behavior, which is one of the major findings of this study. Scholars have pointed out that specific forms of practice, training, and experience can help employees skillfully focus their attention on specific work environments (Hulsheger et al., 2013). At the same time, some scholars have proposed that mindfulness training for employees can help them focus on the present (Dane and Brummel, 2014), improve performance, and reduce employee pressure (Allen et al., 2015), etc. Employees in the new media industry will inevitably meet various high requirements in their work (Dediu et al., 2018). The huge work pressure will have a direct impact on employees’ emotions and work status, and then affect employees’ innovative behavior. Therefore, the management department of the new media industry can improve the mindfulness level of employees in the new media industry through mindfulness training and mindfulness training courses. At the same time, enterprise managers can also incorporate mindfulness into employee training programs and conduct regular training so as to regulate employees’ emotions and work status and lay the foundation for stimulating employees’ innovative behavior.

Secondly, current research results show that positive emotions have a positive effect on improving employees’ innovative behavior. Therefore, enterprise managers can improve employees’ positive emotions by creating an organizational environment that can evoke positive emotions. In the organizational environment, both work and non-work factors may trigger emotional reactions of employees. Therefore, managers in the new media industry can stimulate employees’ positive emotions by creating a relaxed and free working atmosphere, giving positive feedback and encouragement to employees’ work achievements or phased progress, and improving family welfare, and leaders’ care and encouragement. At the same time, they strengthen emotion management ability training, so that new media industry employees can form a stable positive mood, so as to stimulate employees’ innovative behavior.

Finally, work engagement has a positive effect on employees’ innovative behavior, which is another important finding of this study. This finding indicates that managers in the new media industry should attach importance to arousing the enthusiasm of employees. On the one hand, managers can improve the autonomy and enthusiasm of employees by optimizing the work process and content and paying attention to the design of work, enable employees to gain a sense of accomplishment and excitement in the process of work, enhance their enthusiasm and investment in work, and promote their innovative behavior. On the other hand, managers should recognize and encourage employees’ innovative behavior and achievements and provide certain material rewards in addition to spiritual incentives, so as to guide work engagement to produce more innovative achievements.

### Limitations and future research directions

This paper discusses the relationship between mindfulness and innovative behavior among employees in China’s new media industry. Although some valuable research conclusions and practical implications have been obtained, there are still some deficiencies and limitations.

Firstly, the research object of this paper is employees in the new media industry, but employees in different industries have different individual characteristics. Therefore, future research can target employees in different industries to conduct research on mindfulness and innovative behavior. Secondly, convenience sampling was adopted to sample five cities in this study, which was deficient in sample size and sample scope, and the data obtained were all cross-sectional data. In future studies, more effective methods (such as systematic sampling) can be adopted to expand the sample size and sample scope of data collection, and longitudinal studies can also be considered to test the model. Third, although this study verified that positive emotion and work engagement play a chain mediating role in employee mindfulness and innovation behavior, it did not consider whether work engagement also positively affects positive emotion, which can be further verified in future research. Finally, mindfulness has become one of the current research hotspots in human resource management. This study found that employee mindfulness can stimulate or enhance innovative behavior, and future research can start from the leadership level (such as mindful leadership) to explore the mechanism of its interaction with employee innovative behavior.

## Conclusion

The purpose of this study was to investigate the effect of employee mindfulness in the new media industry on innovative behavior. The results indicate that employee mindfulness has a positive impact on innovative behavior. Employee mindfulness positively impacts innovative behavior not only through positive emotion, but also through work engagement. At the same time, this study found that positive emotion and work engagement play a chain mediating role between employee mindfulness and innovative behavior in the new media industry. Therefore, we hope that this study can help enrich the research on mindfulness and innovative behavior of employees in the new media industry and make more contributions to the management of employees in the new media industry.

## Data availability statement

The original contributions presented in the study are included in the article/Supplementary material, further inquiries can be directed to the corresponding authors.

## Ethics statement

Ethical review and approval was not required for the study on human participants in accordance with the local legislation and institutional requirements. Written informed consent for participation was not required for this study in accordance with the national legislation and the institutional requirements.

## Author contributions

TYK proposed research ideas and data collection. YH proposed research framework and data analysis. YL drafted and revised the manuscript. All authors contributed to the article and approved the submitted version.

## Conflict of interest

The authors declare that the research was conducted in the absence of any commercial or financial relationships that could be construed as a potential conflict of interest.

## Publisher’s note

All claims expressed in this article are solely those of the authors and do not necessarily represent those of their affiliated organizations, or those of the publisher, the editors and the reviewers. Any product that may be evaluated in this article, or claim that may be made by its manufacturer, is not guaranteed or endorsed by the publisher.

## Footnotes

^1^ https://www.wjx.cn
